# Perception, Positioning and Decision-Making Algorithms Adaptation for an Autonomous Valet Parking System Based on Infrastructure Reference Points Using One Single LiDAR

**DOI:** 10.3390/s22030979

**Published:** 2022-01-27

**Authors:** Felipe Jiménez, Miguel Clavijo, Alejandro Cerrato

**Affiliations:** University Institute for Automobile Research (INSIA), Campus Sur UPM, Universidad Politécnica de Madrid (UPM), 28031 Madrid, Spain; miguel.clavijo@upm.es (M.C.); a.cerratog@alumnos.upm.es (A.C.)

**Keywords:** valet parking, autonomous vehicle, LiDAR, parking maneuver

## Abstract

Autonomous parking valet systems improve users’ comfort, helping with the task of searching for a parking space and parking maneuvering; and due to the simple infrastructure design and low speeds, this maneuver is quite feasible for automated vehicles. Various demonstrations have been performed in both closed parking and in open air parking; scenarios that allow the use of specific technological tools for navigation and searching for a parking space. However, there are still challenges. The purpose of this paper was the integration of perception, positioning, decision-making, and maneuvering algorithms for the control of an autonomous vehicle in a parking lot with the support of a single LiDAR sensor, and with no additional sensors in the infrastructure. Based on a digital map, which was as simplified as possible, the driver can choose the range of parking spaces in which the vehicle must look for a space. From that moment on, the vehicle moves, looking for free places until an available one in the range selected by the driver is found. Then, the vehicle performs the parking maneuver, choosing between two alternatives to optimize the required space. Tests in a real parking lot, with spaces covered with metallic canopies, showed an accurate behavior.

## 1. Introduction

Vehicle parking is an important problem that has environmental repercussions, causes congestion and time loss for users (and loss of comfort), and that increases in cases such as urban areas. Therefore, various solutions have been suggested, many of them making use of the opportunities that new technologies offer [1]. These solutions involve parking information collection, with various types of sensor, sensor connectivity, and parking system deployment, including software systems or parking vacancy prediction, as well as parking service dissemination. The main challenges for improving parking are classified into different groups, such as sensing, sensor connectivity, data network, data analysis, and connected vehicles, among others. As one of the proposed solutions, the implementation of a valet parking system is becoming popular in areas such as airports [2] or railroad stations [3].

In addition, autonomous vehicles, for parking maneuvers, have advantages with respect to those manually driven. For example, cars could be parked closer together because there would be no need for doors to be opened [4], but this advantage cannot be realized with mixed traffic of autonomous and non-autonomous vehicles. Additionally, if the parking task is done in an automated way, the specific parking space does not matter to the user [5]. Other authors have shown that autonomous vehicle ordering would increase the parking lot capacity by taking advantage of the possibility of blocking one vehicle with another [6], but this solution would have practical limitations, mainly due to user unexpected schedule changes, which would involve frequent and complex vehicle movements.

Related to this idea, automated valet parking systems (AVPS) are being planned that do not have the participation of human drivers. Upon arrival at the parking lot, the human driver leaves the car at a drop-off point. The parking lot is equipped with smart sensor systems that guide vehicles to a parking space. When the driver needs the car, it can be retrieved using a smartphone app, and the car comes to the pick-up point. AVPS improves user comfort, because they do not have to carry out tedious tasks such as searching for a parking space and parking. There is a clear willingness to pay for an autonomous valet system, which was valued at around USD 902, with more than half of surveyed people willing to pay something for the system [7]. In the same paper, a forecast of vehicles with the system implemented in 2025 is presented, and the values oscillate between 13 and 33% approximately, due to the function of the considered scenario.

Although some AVPS solutions have been tested in the past, it cannot be considered a mature technology because some of these solutions rely on detailed digital maps or sensors on the infrastructure, which complicates their widespread applicability. In addition, current vehicle short-range sensors cannot be used for vehicle positioning along the route, only for maneuvering, and the predefined maneuvers require more space than those strictly necessary if they are optimized. This paper deals with the adaptation and configuration of perception, positioning, decision-making, and maneuvering algorithms for a complicated scenario, to optimize performance and improve a real implementation of AVPS. This final system was applied to a parking lot with spaces covered with metallic canopies. This environment presents clear differences, with technological implications in comparison with the experiences in indoor parking and outdoor parking without canopies. Thus, the scenario and approach considered in this paper have very specific characteristics and contributions:

- Outdoor scenarios are more unstructured environments than indoor parking lots, and metallic canopies are more difficult accurately detect than concrete columns.
- However, it is not feasible to monitor free and occupied parking spaces from poles with cameras, due to lack of visibility, and public outdoor parking lots are not equipped with sensors on the canopies. Therefore, the detection must be carried out from the vehicle itself.
- High GNSS positioning accuracy is not guaranteed for these maneuvers, while installation of solutions such as magnetic nails on the ground is not feasible in many cases, and other means of navigation are necessary. Cumulative errors must be reduced or removed.
- It is possible to use prior information from a digital map of the parking lot, but this information must be as reduced as much as possible to foster a widespread implementation, unlike other solutions that require highly detailed maps.

## 2. Related Work

AVPS constitutes one of the applications of a high level of automation according to [8], because it counts on advantages such as reduced speeds and a structured environment. For these reasons, this type of highly-automated vehicle application is one of the most feasible. It should be noted that, already in 2007, automated valet parking was included in a competition in the DARPA Urban Challenge [9]; and later, many initiatives have been launched, such as those planned by BMW or Bosch in collaboration with Ford, or those published in [10,11] for an indoor parking relying on column and wall detection.

The main elements of an AVPS include the environment perception and automation modules of the fully automated vehicle, a mobile phone app as a human–machine interface, and a parking space occupancy detection system based on cameras or short-range sensors in the parking spaces [3]. With this infrastructure, vehicle management and monitoring could be performed [12]. In this line, AVPS can be classified considering the main information source for their operation; computer vision [12], LiDAR [13], and intelligent infrastructure [14] are some of the most common alternatives. In the case of surrounding detection for autonomous navigation, LiDAR has advantages such as not being affected by weather and light, and high accuracy in distance to obstacle estimation.

For vehicle positioning, various methods can be used. Thus, a GNSS/INS receiver with differential corrections is used in [3], as well as SLAM (simultaneous location and mapping) techniques [15]. Highly-demanding positioning must be considered, since the space available for maneuvers is very small; moving the vehicles very close to others and to obstacles. However, a centimetric precision of GNSS receivers cannot be guaranteed for common applications on public roads [16], and parking scenarios are much more demanding than common urban areas, where approaches based on GNSS/INS could work [17]; therefore, specific algorithms are needed for navigation using scenario reference points, avoiding or reducing the impact of potential cumulative errors from INS or SLAM.

Giving the vehicle a priori information about the parking area makes the search more efficient and structured. A widespread solution is to use cameras on a pole and observe the complete parking area for the determination of free spaces. In addition, a communication channel between vehicles and the parking lot could help in managing the available parking spaces. Therefore, almost every AVPS implementation relies on infrastructural intelligence or the pre-acquisition of parking lot digital maps. Thus, [18] shows an approach for deriving a-priori information from available blueprints of a parking lot. In the case of [19], the information required for the parking map is very simple and is limited to coordinates of the parking spaces. An exception, which does not require previous knowledge of the parking lot, is presented in [20].

Despite the above, AVPS involve several open challenges, such as challenging infrastructure (spiral pathway used when changing levels, exiting an indoor parking lot on a sunny day, and some areas with only parallel parking spaces); unpredictable environments, in which vehicles and pedestrians can emerge unexpectedly; challenging environmental conditions, in which rain, snow, or fog reduce visibility; vulnerable road user detection, because pedestrian behaviors are difficult to predict; and accurate short-range detection, to achieve maneuvers in very small spaces [21].

To face these challenges and foster the introduction of autonomous vehicles on to the market, some companies developing AVPS are looking into adapting the infrastructure, and even creating specific robotic platforms as a first step to fully automated valet parking, instead of relying directly on AVPS. Such is the case in [22], where a management solution is proposed for the problems that may arise in these long-period queue congestion systems.

The presented state of the art demonstrates the interest in this technology and the scientific challenges; therefore, the main objective of this paper was the integration and testing of algorithms for perception, positioning, free spaces search, decision-making, and maneuvering, for controlling an autonomous vehicle in an outdoor parking lot with metallic canopies, using a single LiDAR sensor to improve the system performance. Thus, ad-hoc perception algorithms are necessary for detecting canopies, as they are more difficult to detect than columns. Their characteristic elements are used as reference points for vehicle positioning in a simplified digital map, so detection robustness is a must. Finally, parking lot dimensions, driving rules, and vehicle and free parking spaces positions are used in the maneuvering module and two alternatives are considered, unlike in other approaches that are conditioned by a single option. Clear criteria must be defined for choosing one maneuver type and optimizing it.

## 3. System Definition

The proposed system is quite simple for the user, who must only drive to the entrance of the parking lot and select a range of parking places (only if desired because of user choice criteria, or required, for example, in case the vehicle is a rental car and some parking spaces are reserved for the company, or the user is a company employee, and the company has some spaces reserved).

The application in this paper is focused on parking lots with metallic or partially metallic canopies. This infrastructure elements are more difficult to detect than concrete columns in indoor parking lots. Although some of the algorithms are completely generic and could be applied to any parking lot type, reference element detection and vehicle positioning algorithms should be adapted to achieve good and efficient performance in this scenario.

Given the parking characteristics, none of the elements of the system are included in the infrastructure. The complete system is included in the autonomous vehicle and is limited to the following components.

- A LiDAR for surrounding perception. The most convenient location is the vehicle roof, but other locations are also possible if a 360° view is achieved by means of sensor fusion.
- A simplified digital map of numbered parking spaces.
- A user interface, in which the driver selects the group of spaces in which the vehicle should be parked.

Unlike other solutions, such as used in some closed parking lots, this solution does not involve communications between a global management system and the vehicle, since this application is oriented to free open public parking lots, the first vehicle that reaches the space will park in it. However, the system allows for the option of this high-level management, without the need for modifying the whole architecture.

### 3.1. Control Architecture

**Figure 1:**
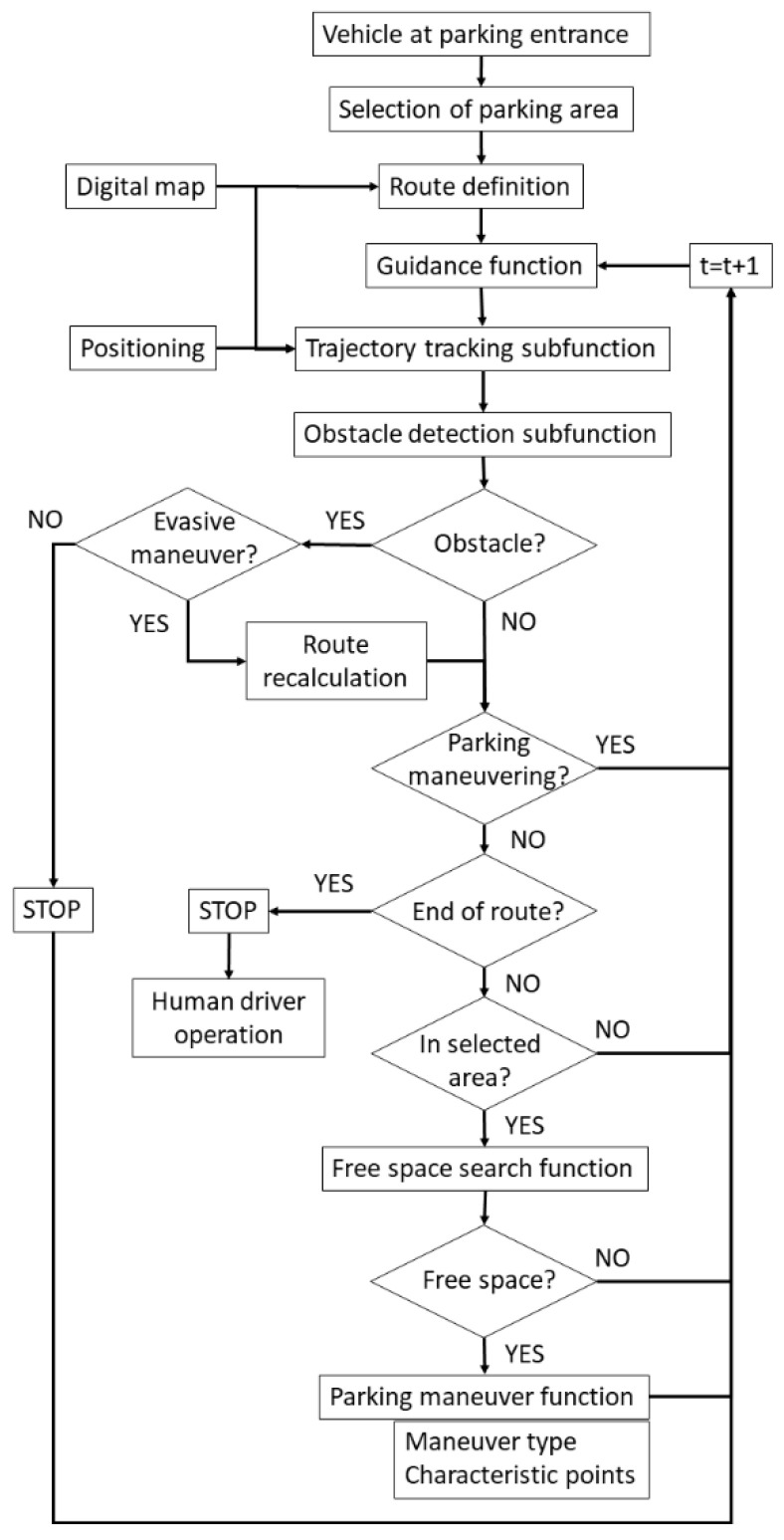
Algorithm of the autonomous valet parking system, including scenario perception, vehicle positioning, guidance function, free space search, and parking maneuver.

The control architecture follows a similar scheme as proposed in [3], but some improvements are included. In this way, since the free spaces are not known a priori, the vehicle must proceed through the parking lot following a route and searching for an available space that meets the conditions set by the user at the beginning. The route to reach that zone is calculated. The *Guidance function* includes the *Route tracking* and *Obstacle detection sub-functions,* which can trigger the vehicle avoidance maneuvers if possible or vehicle detention if necessary (in case of an unavoidable obstacle in the route, the vehicle remains stopped until the obstacle disappears). The first function includes the positioning system on the digital map. When the vehicle is in the desired zone, it looks for free spaces simultaneously with the previous functions. This is the so-called *Free space search function*. Once identified, the parking maneuver type is defined, the vehicle proceeds to the starting point of the maneuver and performs the parking maneuver (*Parking maneuver function,* which sends the orders to the low-level control layer of the autonomous vehicle). In case the whole route is completed without finding a suitable parking space, the vehicle stops at the exit and a human driver must take control again. Figure 1 shows a diagram of the complete algorithm of the autonomous valet parking system.

### 3.2. Parking Digital Map

The car park digital map must contain the numbering of the spaces, so that the user can select the desired range in which the vehicle will search for a free space. This situation is motivated by practical situations, such spaces being reserved for some vehicle types, for workers of a company, or for vehicles of a car rental company. In line with visual positioning solutions [23] and following a similar solution to the one outlined in [19], the map includes only the essential information for planning a route or following a reference element, but in a simplified way and avoiding superfluous data, to encourage easy implementation. Then, the coordinates that delimit the parking spaces with respect to a local reference system are included (it should be noted that absolute positioning is never used). The map also contains geometric information of the limits of the transitable area (walls, curbs, lines), if it is not delimited by parking spaces on both sides, reference points of the main infrastructure elements (canopies and their pillars and roof in the practical application), and driving rules (lanes, allowed directions, etc.). To reduce the map complexity, only geometric information such as reference points coordinates, lines, or curves are included, and the system does not require 3D-representation of the entities, only characteristic coordinates. In this way, in each zone, it is possible to identify the reference elements that condition the navigation of the vehicle. This simplified representation of the parking lot geometry is detailed enough for the system requirements and not very complicated to obtain, considering that these maps are currently not commercially available and very detailed ones would need much more effort and instrumentation.

### 3.3. Route Definition

If the user has introduced a specific zone in which he wants or needs the autonomous vehicle to search for an available place, the algorithm will evaluate the route to reach this area using a shorter distance, respecting any restrictions and prohibited directions. If no restrictions are introduced (because there are no restrictions and the user does not have any preference or a deep knowledge of the parking lot), the system has a preloaded route along the complete parking lot. Although the shortest route may not be the optimal one in some scenarios, in this problem no more criteria for route definition were considered, for simplicity. This fact does not limit the application of the method.

In any case, the algorithm divides the route into zones with different characteristics and stores the elements that serve as a reference in each one of them. Therefore, for example, in one zone, the reference element could be the right curb and, in the next zone, the reference element could change to the left curb. The transition takes place between the final point of the *i* zone and the initial point of the *i +* 1 zone, connected by means of a Bezier curve, as established in [24].

### 3.4. Guidance Function and Trajectory Tracking Subfunction

The perception of the scenario has four fundamental objectives: navigation through the relative positioning of the vehicle on the parking digital map and reference element tracking, obstacles identification to avoid collisions, detection of free parking spaces, and vehicle control during the parking maneuver, to achieve the desired separation with other vehicles and the infrastructure elements (such as canopies pillars or columns).

As previously mentioned, GNSS positioning is not accurate enough, so its use is disregarded, and several studies have shown that visual SLAM techniques imply a non-negligible cumulative error. Therefore, frequent position updating is required, based on reference points whose coordinates are previously known. Although navigation in some zones does not require positioning on the map, because reference elements can be used, local positioning is essential for free space searching and navigation between zones, because the vehicle control layer does not consider physical reference elements detected by the LiDAR. Another reason is the fact that the distance travelled within a zone is required.

The digital map data are stored in local coordinates. Although the coordinates that delimit the parking spaces are included, the perception of these vertices through LiDAR detecting changes in reflectivity is not reliable enough (no clear reflectivity contrast, vehicles covering part of the lines, etc.), so they are discarded as reference elements. Then, accurate (centimetric) positioning in the digital map is a challenge when using one single LiDAR, and reliable reference points must be detected, because global positioning is not a feasible option.

**Figure 2:**
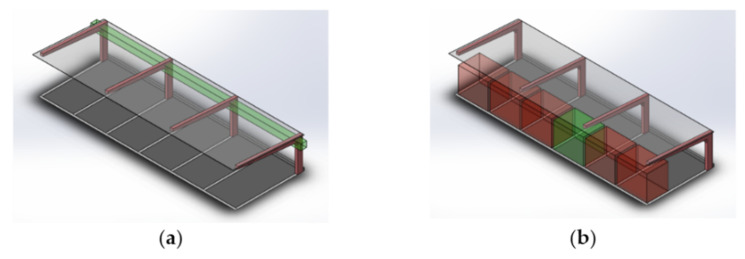
Perception criteria using LiDAR in canopies. (**a**) Procedure for the detection of characteristic points of the canopy (control parallelepiped defined considering the frontal edge of the canopy roof); (**b**) criteria for considering a free parking space (control parallelepiped in red when LiDAR detects obstacles inside, and in green when the volume is free of obstacles and the system estimates that it is a free parking space).

The detection of columns, the upper part of the canopies (pillars and roof), in the specific application shown in the paper, or elements such as curbs is quite reliable. To detect curbs, the algorithm proposed in [25] is used. For the case of the canopies, knowledge of the geometry of their structure is used (Figure 2a). Reference points are taken from the front corners of the roof and the junction points of the pillars and horizontal beams. The frontal limit of the roof is easily detectable, and it is improbable that occlusions will occur that impair the vision. The procedure for locating the remaining points is achieved using this limit line as a reference. In this way, a parallelepiped is defined, whose longitudinal axis extends parallel to the edge of the roof. This axis is in the coordinates of the junction point. Thus, it is possible to determine the position of the canopy pillars where a high density set of points is detected in a section of the parallelepiped. It should be noted that this procedure is based on elements that are not occluded in general, and it is also unlikely that there are elements in the parallelepiped volume that do not belong to the canopy. In the unlikely case of detecting these unexpected elements, correct detection of other pillars and the knowledge of the separation between pillars allow filtering valid data from erroneous data.

Then, the procedure for vehicle positioning is based on the identification of N reference points of the surroundings stored in the digital map. It is possible to determine the number of pillars from the identification of other elements, such as the roof of the canopy, whose limits are easily perceived and provide a reference for the location of the rest of the elements, with respect to the local reference of the digital map. In this way, the error function of Equation (1) is minimized, where (*x_i_*, *y_i_*, *z_i_*) are the coordinates of the *N* reference points detected in each LiDAR frame and *d_i_* is the distance from the vehicle to each of those points. The resolution of the least squares problem provides the coordinates (*X*, *Y*, *Z*) of the vehicle.

(1) $$e^{2}=\overset{N}{\underset{i=1}{\sum }}{\left[{\left(X-x_{i}\right)}^{2}+{\left(Y-y_{i}\right)}^{2}+{\left(Z-z_{i}\right)}^{2}-d_{i}\right]}^{2}$$

**Figure 3:**
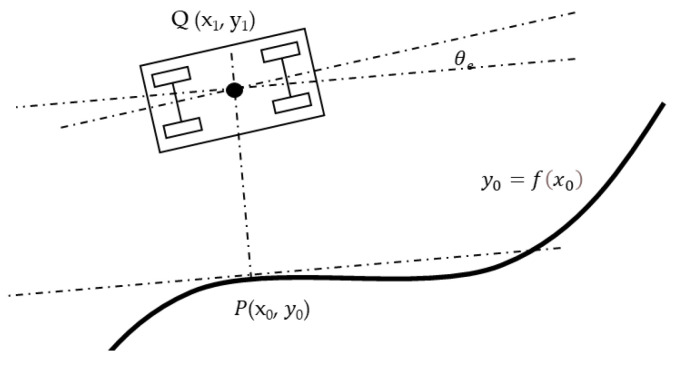
Features considered in the vehicle control algorithm.

For trajectory tracking, in the case of using physical reference elements, as well as the trajectory calculated in the transition stretches between zones, the lateral and angular errors are used [26]. Since the vehicle speed is very low, it is not deemed necessary to introduce any predictive function for the desired path, and the errors between the vehicle position and the projection from this position to the reference curve are computed. Figure 3 shows the main features to be considered in the vehicle control. The orthogonal projection point of the vehicle center Q (*x*_1_, *y*_1_) on the desired route is denoted by P (*x*_0_, *y*_0_). This point must be part of the route, and its normal vector must pass through Q. Therefore, it must verify the equation that represents the reference element given by Equation (2).

(2) $$y_{0}=f\left(x_{0}\right)$$

The perpendicular line to the tangent at P is given by Equation (3) and must pass through the position of the vehicle Q.

(3) $$y_{v}=-\frac{1}{f\prime \left(x_{0}\right)}\left(x_{1}-x_{0}\right)+f\left(x_{0}\right)$$

Knowing P and Q, it is possible to calculate error indicators that serve as control parameters for the vehicle guidance. It is unnecessary to consider the dynamic constraints of the vehicle because of the low speeds, and it is possible to employ a path tracking algorithm based on the Stanley model [27], presented in Equation (4)

(4) $$\delta =\theta_{e}+atan\left(\frac{k\sqrt{{\left(x_{1}-x_{0}\right)}^{2}+{\left(y_{1}-y_{0}\right)}^{2}}}{v}\right)$$

where *θ_e_* represents the angle between the vehicle heading and the tangential direction at P, *δ* is the front wheel angle, *v* is the vehicle speed, and *k* is the gain coefficient.

### 3.5. Obstacle Detection and Free Space Search Functions

The other objectives involve obstacle detection and the implementation of conventional algorithms for the point cloud segmentation [28]. In case of detecting an obstacle, the vehicle will stop if there is not enough free space for an evasive maneuver. Moreover, a parking space will be considered free if there is not an in a parallelepiped that covers the full entrance to the space and whose length is one third of the total space length (Figure 2b). This criterion does not prevent the detection of false free spaces, but its motivation is based on the difficulty of perceiving with LiDAR the whole space early enough to carry out the parking maneuver. In case the space has been wrongly detected as free, the system will reconsider this classification during the maneuver and will abort and continue its search.

### 3.6. Parking Maneuver Function

In the parking process, the vehicle speed is very low, so it is unnecessary to consider the dynamic constraints of the vehicle, and geometric considerations can be applied. In this way, it is necessary to consider the position of the instantaneous rotation center in the extension of the vehicle rear axle and assume that the front wheels fulfil Ackerman’s geometry, so that no slip angles are produced [29].

In this system, only perpendicular parking maneuvers entering the space in reverse gear are considered. Reference [30] includes a general compilation of algorithms that are used for trajectory planning. More specifically, in the case of [19], the geometric calculation of the parking maneuver is planned using an advanced position of the vehicle in a perpendicular orientation to the space and without changing direction in the maneuver. Two cases were studied: a simple approach of a constant radius, and the generalization with clothoid sections at the beginning and end of the turning of the steering wheel, to consider variable steering wheel angles during movement, and not only changes in those angles when the vehicle is stopped. In [12] a maneuver geometry was also built with circumferential arcs, but a new approach is introduced considering tangent circumferences, with a change from forward to reverse between both phases. This solution has an advantage compared to the previous one, in that the starting point of the maneuver is closer to the parking spaces and the intrusion into adjacent lanes is smaller. Other alternatives, not based on circumferences, are found in [3], which uses rational Bézier curves for the definition of the trajectory. In the same line, [10] proposes a solution based on splines.

Although Bezier curves and splines are mathematically very satisfactory and the first solution is used in the definition of the theoretical path in the transitions between zones with different reference elements, in the case of the parking maneuver, more intuitive solutions are preferred, and so constant turns during the main phase of the maneuver were chosen (trajectory built from circumferences and straight stretches). Common approaches use a single maneuver type for solving the problem. However, spatial restrictions in parking lots represent a challenge, and maneuvers must be optimized, guaranteeing safety. In this case, two types of maneuvers are defined, and two approaches included in the state of the art are now integrated, to allow use of the most convenient one in each scenario:

- Maneuver I: when the transversal separation of the vehicle to the parking spaces is greater than a certain value, the approach of [19] is used.
- Maneuver II: when the distance is smaller than a certain value or some maneuverability factors are not fulfilled, the approach of [12] is used.

**Figure 4:**
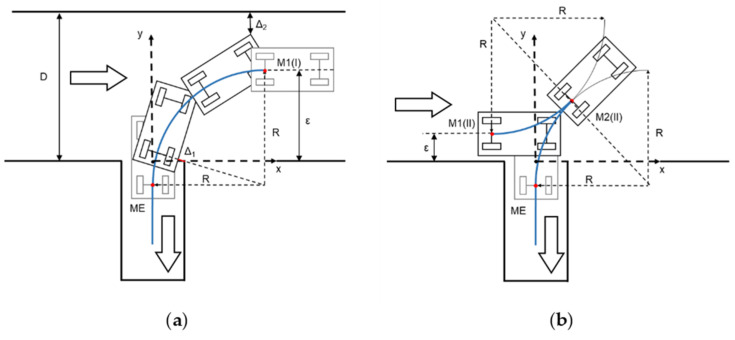
Diagrams for calculating the relevant points of the parking maneuver. (**a**) Maneuver I; (**b**) Maneuver II.

This approach takes advantage of the most convenient maneuver, depending on the scenario, and it is not confined to one option, as in previous works. Figure 4 shows the relevant points of both maneuvers.

In the calculations of both maneuvers, the procedure starts from the key point on which the entire geometry of the maneuver is built: the ME point of entry to the parking space, which must be reached by the vehicle with longitudinal orientation and without having interfered with the adjacent spaces (with a safety margin Δ_1_ being respected). From this point on, the vehicle must carry out transversal control, following the space limits and adjacent vehicles. In addition, it is assumed that the trajectory center of rotation is placed on the prolongation of the vehicle rear axle line. Taking the center point of the entrance to the parking space as the coordinate origin, the coordinates of the ME point are given by Equation (5).

(5) $$\mathrm{ME}:\;\left(0,-\sqrt{{\left(R-\frac{W_{v}}{2}-\Delta_{1}\right)}^{2}-{\left(R-\frac{W}{2}\right)}^{2}}\right)$$

where *W* is the parking space width, *W_v_* is the vehicle width, *R* is the radius of the rear axle trajectory, and Δ_1_ is a safety distance regarding the non-intrusion into the adjacent parking space. It should be noted that the minimum value of *R* may differ from one vehicle to another, so generic calculations must consider a conservative value suitable for all (or most) passenger cars. In general, *R* can be estimated between 4 and 8 m. In a more customized solution, calculations for each vehicle could adapt them to the real values.

The starting point of the maneuver I, called M1(I), is derived from Figure 4a and is given by Equation (6).

(6) $$M1(I):\;\left(R,\;R-\sqrt{{\left(R-\frac{W_{v}}{2}-\Delta_{1}\right)}^{2}-{\left(R-\frac{W}{2}\right)}^{2}}\right)$$

Finally, it must be verified that the corridor width is large enough for completion of the maneuver, so that the vehicle front overhang distance *L_fv_* does not exceed its limits, so Equation (7) is verified:

(7) $$D-\left(\sqrt{{\left(R+\frac{W_{v}}{2}\right)}^{2}+{\left(L_{v}+L_{fv}\right)}^{2}}-\sqrt{{\left(R-\frac{W_{v}}{2}-\Delta_{1}\right)}^{2}-{\left(R-\frac{W}{2}\right)}^{2}}\right)\geq \Delta_{2}$$

where *D* is the corridor width, *L_v_* is the distance between the vehicle axles, and Δ_2_ is the safety distance to be guaranteed. It should be noted that, although they can be configured in each case, and safety margin Δ_1_ and Δ_2_ can be set to around 0.3–0.5 m.

If the transversal distance *ε* between the vehicle and the spaces is greater than the Y-coordinate of M1(I) point, maneuver I can be performed by displacing ME and M1(I) points according to the axle Y, the same distance as the difference between *ε* and Y(M1(I)). The maximum admissible distance *ε* is given by Equation (8).

(8) $$\varepsilon_{max}=R+D-\Delta_{2}-\sqrt{{\left(R+\frac{W_{v}}{2}\right)}^{2}+{\left(L_{v}+L_{fv}\right)}^{2}}$$

If the initial value of the distance is greater than the one given by Equation (8), it must be reduced by this maximum value, so that Bezier curves are used in the same way as in the transitions between zones. In this case, if it is still greater than the coordinate Y(M1(I)) calculated in Equation (6), maneuver I can be performed. Otherwise, maneuver II must be performed.

In the situation of Figure 4b, it is not necessary to complete the maneuver of a circumferential sector of 90°, as the starting point of the maneuver is replaced by a new starting point M1(II) and a change of direction point M2(II). The coordinates of these points are given by Equations (9) and (10):

(9) $$M1(\mathrm{II}):\;\left(\sqrt{{\left(2R\right)}^{2}-{\left(\varepsilon +R+\left|Y\left(\mathrm{ME}\right)\right|\right)}^{2}}-R,\varepsilon \right)$$

(10) $$M2(\mathrm{II}):\;\left(\frac{1}{2}\left(X\left(M1\left(I\right)\right)+X\left(M1\left(\mathrm{II}\right)\right)\right),\sqrt{R^{2}-{\left(X\left(M1\left(\mathrm{II}\right)\right)\right)}^{2}}-\left|Y\left(\mathrm{ME}\right)\right|\right)$$

**Figure 5:**
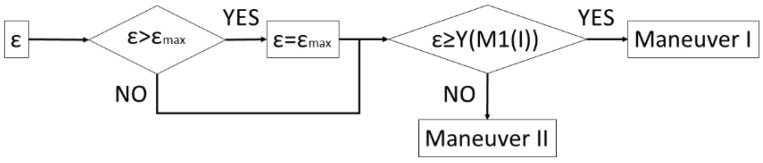
Car parking maneuver type decision algorithm.

In summary, Figure 5 shows the decision logic for the selection of the maneuver type. At the first check point, it is verified whether the transversal vehicle position has to be corrected to be able to complete the parking maneuver in the D corridor space. The second check is the one that distinguishes the type of parking maneuver.

In any case, it is important to note that these maneuvers are calculated at a theoretical level and represent a target path for the autonomous vehicle, but adjustments must be carried out in real time, taking into account the monitoring of the limits of the space in the functioning of the information of the onboard sensors (position of the canopy poles, detection of spaces lines if there is enough reflectivity contrast, distance from adjacent vehicles).

## 4. Tests

### 4.1. Tests Definition

**Figure 6:**
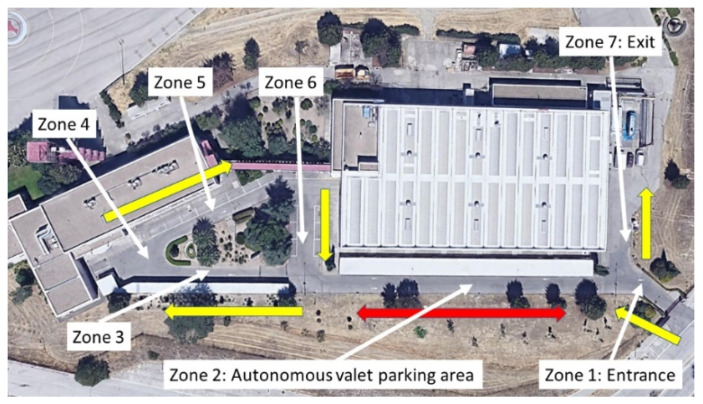
Parking area of University Institute for Automobile Research.

The system was tested in the parking area of the facilities of the University Institute for Automobile Research of the Technical University of Madrid (Spain). This parking area includes a covered parking area with a canopy for perpendicular parking and another one for parking inline, both covered and uncovered. The autonomous valet parking system was designed exclusively for the first spaces. Moreover, they are close to a high building that would reduce GNSS signal accuracy. Figure 6 shows an aerial view of the parking area, in which different zones are defined. In this parking lot, all the streets are one-way, except for the straight lane in zone 2 with the spaces for the AVPS. Zone 2 is two-way street, and it was used to simulate two scenarios:

- (S1) It is considered that the vehicles in this area can coincide in this area, whereby the vehicles circulate along the center of the section (they can park in any of the two directions).
- (S2) This zone is considered to have two lanes, one in each direction, and the vehicles move using the correct lane (strictly, the vehicle can only park if the parking space is adjacent to the lane, unless expressly authorized).

Likewise, Table 1 includes the infrastructure elements that serve for vehicle navigation in each area.

**Figure 7:**
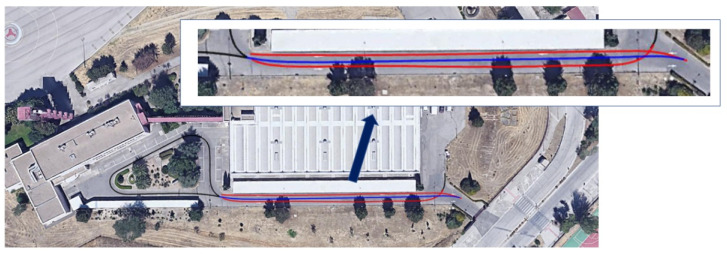
Vehicle trajectories in the parking lot (blue: S1; red: S2; black: common).

Figure 7 shows the complete route of a vehicle from the entry zone to the exit zone, in case a valid place is not found, where the only difference appears in zone 2. It should be noted that only one trajectory is possible, because of the configuration of the streets of the parking lot, so the algorithms do not have to choose a route from among several alternatives.

Finally, the vehicle must have the ability to deal with any combination of the following cases when entering the parking lot:

1. Initial parking space group selection by the driver (which conditions the search route):
  - No selection/range selection.
2. Availability of parking spaces in the selected group:
  - No available spaces (the vehicle must finish at the exit zone)/only one available space/more than one available space (the first one must be occupied).
3. Availability of parking spaces outside the selected group (these spaces must be discarded by the system in the search operation):
  - No available spaces/available spaces.
4. Circulation scenarios and free space identification:
  - Case A: Scenario S1 driving along the center of zone 2 stretch (parking maneuver moving from 1 to 3).
  - Case B: Scenario S1 driving along the center of zone 2 stretch (parking maneuver moving from 6 to 7).
  - Case C: Scenario S2 driving using the right lane (parking maneuver moving from 1 to 3).
  - Case D: Scenario S2 driving using the right lane (parking maneuver moving from 6 to 7, with prior authorization).

**Figure 8:**
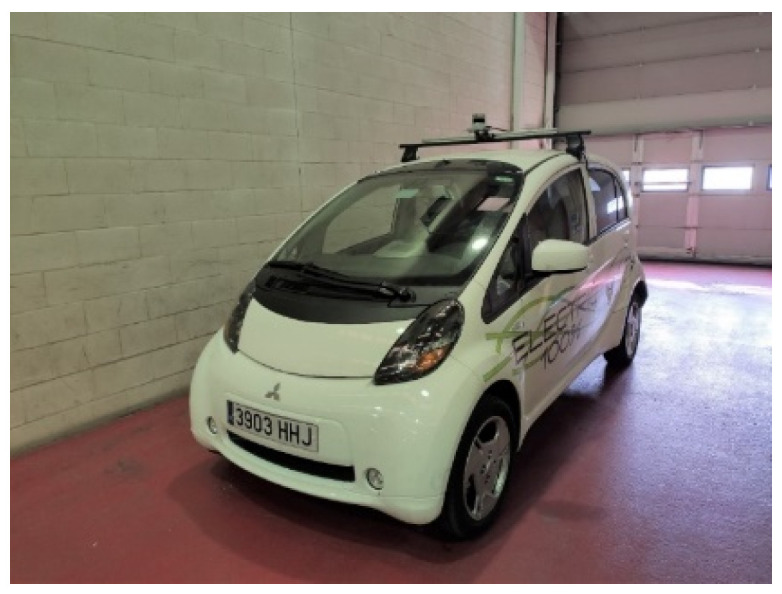
Autonomous vehicle used in tests.

For the tests, a Mitsubishi Imiev autonomous vehicle was used, equipped with an Ouster OS-1 64-layer on the roof (Figure 8), which was used for positioning and detection of parking spaces. Additionally, to check the maneuvers, but not used for the vehicle control, a GNSS receiver was included. It should be noted that this positioning source was not accurate enough for most stretches of the trajectory, and its use was only informative and qualitative.

### 4.2. Tests Results

Every possible scenario in the system functioning was considered and prepared. Although improvements were focused on algorithms in some parts of the system, their isolated performance could not be verified in a quantitative way, or a reliable ground truth was not available. For this reason, the correct performance of the algorithms was verified by looking at the whole system performance.

**Figure 9:**
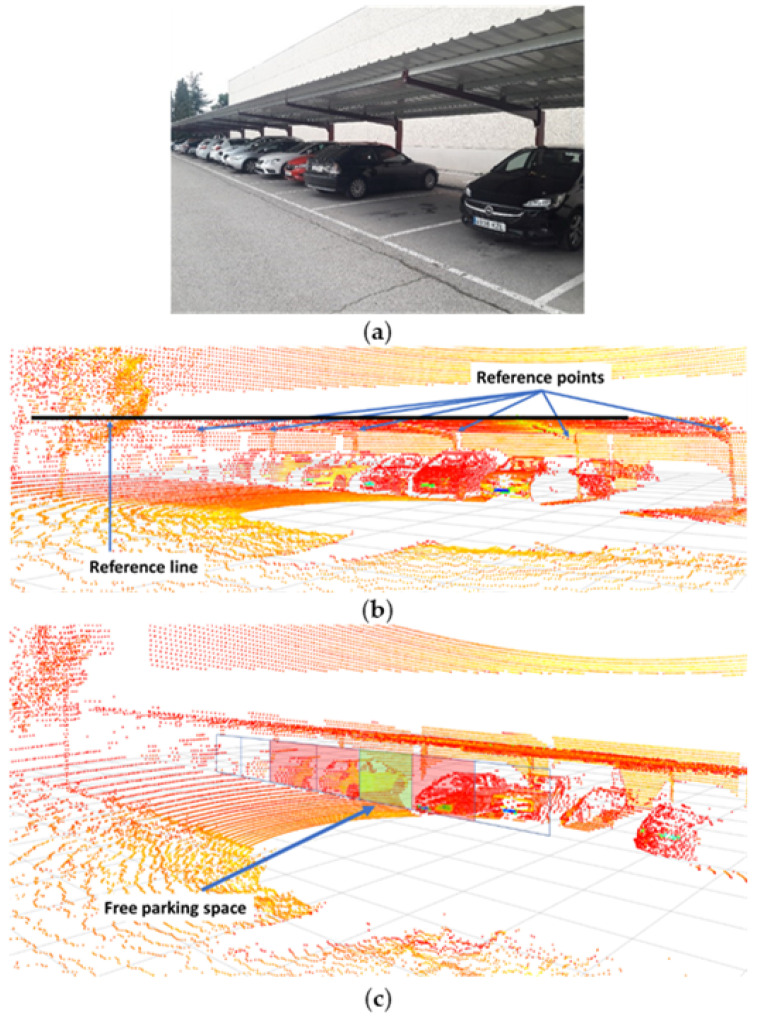
Detection of reference elements and free and occupied parking spaces. (**a**) Scenario; (**b**) reference element detection; (**c**) free parking spaces detection.

The first three possible case combinations cited above (spaces group selection, availability of parking spaces in the selected group and outside it) were verified in a qualitative manner in the tests set. It was observed that the system chose the correct decision in every case, and the appropriate parking space was selected, or the route until the exit zone was performed if necessary. In addition, the guidance function provided a good performance, without severe oscillations (registered lateral and angular errors are lower than 0.1 m and 5.4°, respectively). Figure 9 shows the detection of the main elements of the canopy and the detection of free and occupied parking spaces using methods and criteria presented in Section 3.4 and Section 3.5. The border line of the canopy roof was considered as the main reference for the positioning of the other elements and helped in the location of the junction points of pillars and horizontal beams. Then, with this information, parking spaces were estimated between pillars and their occupancy was analyzed. The challenges of local vehicle positioning and guidance along the parking lot were overcome with a single LiDAR, which looked for very specific reference elements, and a simplified digital map.

**Figure 10:**
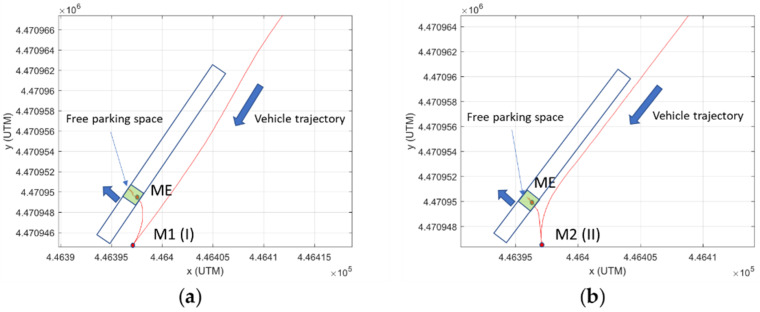
Trajectories during parking maneuvers. (**a**) Maneuver type I in Case A; (**b**) Maneuver type II in Case C.

**Figure 11:**
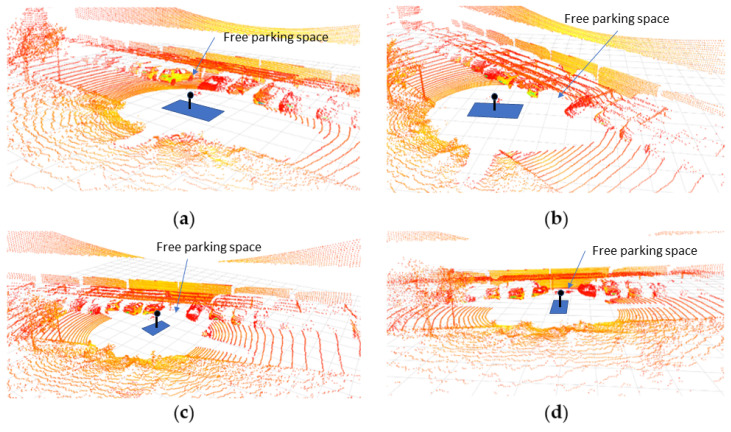
Example of a sequence of LiDAR frames during the parking maneuver in Case A. (**a**) Approximation; (**b**) reverse maneuver between M1(I) and ME; (**c**) reverse maneuver near ME; (**d**) final straight maneuver.

Finally, the four circulation scenarios defined above are considered. Table 2 shows the vehicle and parking lot data, as well as the calculations of the parameters for choosing the optimal maneuver type. If in zone 2, the vehicle is moving along the central line (S1), the type I parking maneuver is chosen by the system. This maneuver would be chosen both, starting at the entrance (from zone 1 to 3, case A) or going to the exit zone (from zone 6 to 7, case B). This decision is taken based on the condition that ε_max_ = 4.3 m > ε = 3.2 m > Y (M1(I)) = 3.1 m, according of the algorithm in Figure 5. If in zone 2, the traffic senses are respected (S2) and a free space is found when moving from the entrance (from zone 1 to zone 3, case C), parking maneuver II is performed because ε = 1.6 m < Y (M1(I)) = 3.1 m. Then, the starting point of this maneuver is closer to the parked vehicles and the intrusion on the opposite lane is smaller than the case of maneuver I. In the case where a free space is detected when moving towards the exit (case D), maneuver type I is chosen, and the vehicle must adapt the transversal distance ε in the approximation maneuver because ε = 4.8 m > ε_max_ = 4.3 m. Table 3 presents the main points of the parking maneuver in each case. Figure 10 shows the vehicle trajectories in the UTM (universal transverse Mercator) Cartesian coordinates system in both tests: parking maneuver I in Case A and maneuver II in Case C. It should be noted that the GNSS positioning was not accurate below the canopy, as expected, and some noise could be discerned in the signal, so the uselessness of this signal for control purposes is clear, and the proposed positioning method is justified. For the same reason, this GNSS signal is not feasible for positioning ground truth. Figure 11 shows a sequence of LiDAR perceptions during the parking maneuver in the case of the maneuver in case A.

The tests demonstrated the accurate performance of the system, which integrates the algorithms for scenario perception, vehicle positioning using infrastructure elements, decision-making, vehicle guidance along the route, and the parking maneuver (scenarios that involved the two types of maneuver were tested). The results showed the robustness of the detection of reference elements of the canopies, but the system requires the use of a digital map in those stretches between zones with different reference elements and for parking space searching. This dependence of the system on a digital map is the main limitation to the widespread implementation of the system. Finally, the system does not consider the interaction with other vehicles to optimize performance, but only evasive or stopping maneuvers are considered in case of conflict.

## 5. Conclusions

This paper presents a valet parking system for autonomous vehicles in a scenario where the technologies used for this type of application are not completely valid. Specifically, a public surface parking lot with metallic canopies covering spaces was considered; however, some of the integrated algorithms are valid for any parking lot type. This scenario did not take advantage of the benefits of uncovered parking lots or underground car parks, which were used in the solutions referenced in the state of the art. In this way, it is not possible to use sensors for supervising the state of locations from the infrastructure (with sensors in their own locations, or cameras in elevated positions), and onboard sensors in vehicles must be used. Moreover, in this type of parking lot (and, in general, for this type of maneuver in a very restricted space), GNSS positioning may not be accurate enough. For this reason, navigation is carried out exclusively using LiDAR technology and using parts of the canopy as a reference element.

Some of the algorithms that integrate AVPS were adapted and configured for a complicated scenario, to optimize performance and improve real implementation. In this sense, detection of metallic or partially metallic canopies is more difficult than concrete columns in indoor parking lots, but a specific algorithm was developed to overcome this problem and obtain a reliable and robust perception and vehicle relative positioning. In addition, the system was based on knowledge of a digital parking map. This map includes little information (coordinates of reference elements, parking spaces, and canopies), unlike other applications with highly detailed maps that provide references and additional aids for maneuvering, which will not be available for widespread implementation in the near future. Finally, with the aim of optimizing the maneuver, special attention was paid to the starting point of the maneuver, defining possible cases. In each scenario, the system chose the most convenient maneuver option, calculating the stopping points, and this approach improved the system performance in comparison with other systems that only consider one maneuver type, and which may encounter limitations because of the parking lot dimensions.

The system was tested with an autonomous vehicle in a parking lot that included spaces that met the specified conditions, while those that did not meet them were declared as not valid for the valet parking operation. The results showed correct functioning in all the scenarios and when performing the two types of maneuver.

It should be noted that the sensors and automatic parking systems which are already fitted on some cars were not considered in this work, and the entire solution was based on ae single LiDAR. Existing sensors in parking systems (mainly, short range sensors) could play a supporting role in the parking maneuver, but not in the other tasks of the presented system, such as positioning or guidance of the free space search.

The results show the following contributions that overcome the main challenges for optimizing the system:

1. A single LiDAR provides enough information for positioning, guidance, and parking maneuvers; therefore, the vehicle equipment is simplified and no additions to the infrastructure are necessary. The specific algorithms for the physical scenario (e.g., detection of canopies elements) improved the performance.
2. The information in the digital map was substantially reduced, so their construction is easy and widespread implementation would be faster.
3. Limited space in parking lots is a common problem for these kinds of automatic systems, but the option of choosing between two maneuver types and the criteria for this selection and the definition of main reference points make the system more efficient.

Finally, the procedure described and tested in this paper could be generalized to other parking lots, such as covered ones, but the reference elements for positioning would have to be adapted. However, as indicated above, it is considered that these cases may prove easier, given the simpler structure of indoor parking lots, the existence of easily detectable elements such as walls or columns, and the possibility of incorporating sensors into the infrastructure. The modular construction of the software allows changes and improvements to specific functions. Some future work involves more complex algorithms for predefining the initial route for parking space searching, cooperation between vehicles, and global high-level management for optimizing performance in a global way and increasing of perception intelligence, in order to reduce the dependency on the digital map and reduce the amount of information it contains. More accurate, realistic, or customized parking maneuvers could also be considered, but the impact of such changes would be negligible, because the control algorithm used for vehicle guidance absorbs small deviations.

## Figures and Tables

**Table 1:** Reference elements for vehicle guidance.

	Reference Element for Autonomous Navigation	
Zone	Main	Secondary
1	Left curb	-
2	Left curb	Right canopy
3	Right curb	Left canopy
4	Right curb	-
5	Right curb	Vehicles/left curb
6	Building wall	Vehicles/right curb/left curb
7	Right curb	-

Transitions between zones 2–3, 5–6, 6–2, and 2–7 are completed by Bezier curves.

**Table 2:** Vehicle and parking lot data, and parking maneuver function parameters for the maneuver type choice.

Vehicle Data (m)		Parking Lot Data and Safety Margin (m)		Parking Maneuver Function Parameters (m)	
Lv	2.5	D	6.4	ME	(0; −0.9)
Lfv	0.8	W	2.5	M1(I)–theoretical	(4.0; 3.1)
Wv	1.6	Δ1	0.3	ε_max_	4.3
R	4.0	Δ2	0.3		

**Table 3:** Main point coordinates of the parking maneuver.

	Cases A, B		Case C		Case D	
**ε (m)**	3.2		1.6		4.8	
**Maneuver**	Type I		Type II		Type I	
**Coordinates (m)**	M1 (I)	(4.0; 3.2)	M1 (II)	(0.6; 1.6)	M1 (I)	(4.0; 4.3)
			M2 (II)	(2.3; 3.0)		
